# Diversity of Aerobic Bacteria Isolated from Oral and Cloacal Cavities from Free-Living Snakes Species in Costa Rica Rainforest

**DOI:** 10.1155/2017/8934285

**Published:** 2017-08-20

**Authors:** Allan Artavia-León, Ariel Romero-Guerrero, Carolina Sancho-Blanco, Norman Rojas, Rodolfo Umaña-Castro

**Affiliations:** ^1^Laboratorio de Análisis Genómico, Escuela de Ciencias Biológicas, Universidad Nacional, 86-3000 Heredia, Costa Rica; ^2^Laboratorio de Biología Molecular, Universidad de Costa Rica, Sede del Atlántico, Turrialba, Costa Rica; ^3^Centro de Investigación en Enfermedades Tropicales, Facultad de Microbiología, Universidad de Costa Rica, San José, Costa Rica

## Abstract

Costa Rica has a significant number of snakebites per year and bacterial infections are often complications in these animal bites. Hereby, this study aims to identify, characterize, and report the diversity of the bacterial community in the oral and cloacal cavities of venomous and nonvenomous snakes found in wildlife in Costa Rica. The snakes where captured by casual encounter search between August and November of 2014 in the Quebrada González sector, in Braulio Carrillo National Park. A total of 120 swabs, oral and cloacal, were taken from 16 individuals of the Viperidae and Colubridae families. Samples were cultured on four different media at room temperature. Once isolated in pure culture, colonies were identified with the VITEK® 2C platform (bioMérieux). In order to test the identification provided on environmental isolates, molecular analyses were conducted on 27 isolates of different bacterial species. Specific 16S rDNA PCR-mediated amplification for bacterial taxonomy was performed, then sequenced, and compared with sequences of Ribosomal Database Project (RDP). From 90 bacterial isolates, 40 different bacterial species were identified from both oral and cloacal swabs. These results indicate the diversity of opportunistic pathogens present and their potential to generate infections and zoonosis in humans.

## 1. Introduction

Costa Rica is one of the countries with the highest rates of biodiversity per km^2^. It includes 143 species of snakes described. Within them, some species are venomous and potentially life-threatening to animals and humans: five species of coral snakes (family Elapidae, subfamily Elapinae), sixteen of pit vipers, such as* Bothrops asper *and* Bothriechis schlegelii *(family Viperidae, subfamily Crotalinae), and a sea snake (family Elapidae, subfamily Hydrophiinae) [1].

The family Colubridae, considered as nonvenomous, holds approximately 104 species like* Sibon longifrenis *(subfamily Dipsadinae) and* Oxybelis brevirostris* (subfamily Colubrinae) [2, 3].

In tropical regions, snakebites are important health problems [4]. Only in Central America, approximately 4000 snakebites have been registered per year, being the agricultural workers and/or rural residents the most affected [5, 6]. In Costa Rica, the average of snakebites was 504 reports per year during 1990 to 2000 [7]. The study of these animals has focused greatly on poisonous species for their medical interest, since they are responsible for a significant number of snakebite incidents in the country [8].

During the period 1990–2000, a total of 5550 snakebite accidents were reported in hospitals and other health centers in Costa Rica. High variation was observed in the number of cases per year, ranging from 423 (1999) to 590 (1992). No trend was observed in the absolute number of snakebites over time averaging 504 reports per year [7]. However, the bacterial infections are often secondary complications of wounds to animal bites, and it has been determined that pathogenic microorganisms responsible for infection are also present in the oral flora of the biting animal [9, 10].

It has been established that the ingested diet and its oral flora directly influence the oral microbiota of the snakes. It has been proposed that cloacal flora of the prey animals can be found in the oral cavity of the snakes, due to the prey defecating by the time it is ingested [9, 11]. Despite the influence of associations of bacteria and snakes and the influence of these bacteria on humans, there are few studies on the characterization and distribution of these microorganisms [12]. However, some bacterial distributions, including some Gram-positive bacteria such as* Staphylococcus *sp., have been confirmed in snakes biota. They are predominant in the oral cavities of healthy snakes but Gram-negative bacteria such as* Pseudomonas aeruginosa, Providencia rettgeri, *and* Pseudomonas maltophilia* (currently* Stenotrophomonas maltophilia*) are predominant in the oral cavities of snakes with stomatitis [12, 13]. Other members belonging to the family Enterobacteriaceae can cause respiratory diseases in humans [12]. Also some species of the genus* Stenotrophomonas *sp., for example,* S. maltophilia,* may induce diseases such as endocarditis, sepsis, meningitis, peritonitis, soft tissue infections, and wounds [14].

The aim of this study is to identify, characterize, and report the diversity of the bacterial community in the oral and cloacal cavities of venomous and nonvenomous snakes found in wildlife into the rainforest of central volcanic mountain range, Costa Rica.

## 2. Material and Methods

### 2.1. Area of Study

The* Quebrada González *sector forms part of the vast Braulio Carrillo National Park located in* 10*°*09*′*39.88*′′*N y 83*°*56*′*13.97*′′*O*. The forest located in the area is montane rainforest transitioning to tropical moist basal. It has been registered up to 6375,5 mm of annual precipitation and an average temperature of 24°C. On the other hand, the site's average elevation is 514,4 ± 81,3 m and has strong inclines in most of its area [15]. The forest composition varies from secondary to mature forests, including open areas formed by the Sucio river [15–17]. Also present are various important tributaries like Quebrada González that have a constant flow throughout the year, making the water source abundant in the sector [18, 19].

### 2.2. Sample Collection

The snakes were captured between August and November, 2014. On each field trip, sampling took place in the morning (7 a.m.–11 a.m.) and at night (7 p.m.–11 p.m.); since there are more active individuals [2, 19], search was conducted with an intensive search technique for a casual encounter [20], looking for individuals on the ground, on leaf litter, and on top of vegetation. Snakes were captured with the assistance of herpetological tongs and identified to a species level. A total of 120 swabs, oral and cloacal, were taken from 16 individuals of both Viperidae and Colubridae families. Samples were taken and immediately cultured on four different media at room temperature; Mannitol-salt agar (MSA), MacConkey agar (MCA),* Salmonella-Shigella* agar (SSA), and blood agar (BA). Cultures were taken to the laboratory on a 24- to 48-hour period; bacterial isolates were separated by morphology and Gram staining. Once grown and pure, cultures were inoculated on blood agar or trypticase soy agar for further processing. After 24 h, these isolates were taken to the Laboratorio de Bacteriología Médica, Facultad de Microbiología, Universidad de Costa Rica, to be identified and submitted to antibiotic susceptibility testing (AST) with the VITEK 2C platform (bioMérieux). To verify the identification provided on environmental isolates, molecular analyses were conducted on 27 isolates of different bacterial species.

### 2.3. Total DNA Extraction and PCR

Total genomic extraction was performed of bacterial pellets after strong centrifugation using STES buffer (0.2 M Tris-HCL, 0.5 M NaCl, 0.01 M EDTA, 1% SDS) and standard phenol/chloroform extraction method was performed [21]. Specific 16S rDNA PCR-mediated amplification for bacterial taxonomy was executed with the following primers: 5′-AGAGTTTGATCMTGGCTCAG-3′ and 5′-GTTACCTTGTTACGACTT-3′ [22]. Polymerase chain reactions were arranged at a 20 *μ*l final volume with PCR Master Mix (2x) (Thermo Scientific®), 0.3 *μ*M forward and reverse primer, and 100 ng of bacterial genomic DNA template. Thermocycling parameters for the gene fragment amplification consisted of initial denaturalization at 95°C for 5 min, followed by 36 cycles of denaturalization (95°C, 45 s), annealing (58–62°C, 1 min, 30 s), extension (72°C, 1 min, 15 s), and final extension step at 72°C for 7 min. PCR reactions were conducted on a thermal cycler (Proflex PCR System; Applied Biosystems, Life Technologies, USA). Amplicons were visualized by agarose gel electrophoresis (1.5%) in TBE 1x (Tris-base, boric acid, EDTA, pH 8), stained with GelRed® (Biotium). GeneRuler 1 kb plus DNA Ladder (Thermo Scientific) was used as size marker. Molecular biology grade water (Ambion®) was used as negative control.

### 2.4. Sequencing and Bioinformatics Analysis

PCR products were purified by isopropanol precipitation and quantified with a NanoDrop 2000 spectrophotometer (Thermo Scientific) and used for direct DNA sequencing. Partial gene fragments of 16S rRNA were sequenced, using the same amplification primer (BigDye Terminator® V3.1, Applied Biosystems), according to manufacturer's instructions. The resulting products were purified with the Xterminator Kit (Applied Biosystems) and then run on DNA multicapillary sequencer (Model 3130, Applied Biosystems) at the Laboratorio de Análisis Genómico, Escuela de Ciencias Biológicas, Universidad Nacional, Costa Rica. Recovered sequences were edited using Geneious® R9 version (Biomatters Ltda), analyzed with BLASTn algorithm [23] at the NCBI (http://www.ncbi.nlm.nih.gov/blast) with the 16S ribosomal DNA sequences (Bacteria and Archaea) database, and compared with other previously published sequences. On the other hand, all bacterial 16S ribosomal DNA sequences obtained were compared with sequences of Ribosomal Database Project (RDP) database using the Seq Match algorithm, parameter S_ab score (http://rdp.cme.msu.edu/) for sequence similarities searches to confirm bacterial identity [24]. Our nucleotide sequence data for 16S rRNA gene was deposited in GenBank (https://www.ncbi.nlm.nih.gov/genbank/) under accession numbers KY963324 to KY963344.

Local sequences and ones obtained at GenBank database were dereplicated by USEARCH v7.0 software [25] through cluster fast command application (under a threshold identity 0.99000). Then, nonduplicates clusters sequences were aligned using MUSCLE algorithm [26] with default parameters. Phylogenetic tree was performed using maximum likelihood (ML) by raxmlGUI v.7.4.2 [27, 28] software through GTR-GAMMA substitution model and 1000 rapid bootstrap inferences. The consensus trees were visualized and edited in FigTree 1.4 program [29].

## 3. Results 

A total of 90 bacterial isolates (from 120 cloacal and oral swabs) were recovered from 16 individuals of several species including* Bothrops asper, Bothriechis schlegelii, Leptodeira septentrionalis, Sibon longifrenis, Oxyrhopus petolarius, Oxybelis brevirostris, *and* Imantodes cenchoa. *Overall 40 different bacterial species (12 families) were identified by VITEK approach from both oral and cloacal swabs (Table 1). Both Viperidae species,* B. asper* and* B. schlegelii, *isolates had the most different bacterial morphotype with 32 and 18, respectively, followed by* S. longifrenis *with 13,* O. petolarius *with 9,* L. septentrionalis *and* I. cenchoa *with 7, and finally* O. brevirostris *with 4 isolates (data not shown). About the distinctive colony phenotypes, 47 of the isolates were found in oral swabs and 43 on the cloacal swabs. From all the isolates identified with the VITEK platform, none are certainly exclusive from either oral or cloacal swabs. However, the few isolates that were identified as a unique bacterial species in either cavity were identified only once. Amongst these unique genera, we can find* Bordetella, Salmonella, Elizabethkingia, Sphingomonas, *and* Rhizobium*.

In the family Enterobacteriaceae, a general resistance pattern was found for ampicillin and cephalothin, being susceptible to these antibiotics* E. coli *and* R. ornithinolytica, *respectively. On the other hand, they were widely susceptible to various antibiotics: PpC/Tzba, Cfa, Cftz, Cfe, Imi, Mer, Ami, Gen, and Cip2. The only exception is* H. alvei *showing resistance to the combination of PpC/Tzba. The second family with more representatives in our AST was Staphylococcaceae. It was widely susceptible to most of the antibiotics; however,* S. saprophyticus *and* S. warneri *were the only species with resistance to antibiotics. Additionally, several bacterial isolates identified as opportunistic pathogens show resistance to different antibiotics, for example,* Aeromonas hydrophila* (Amp and Amp/Sbt),* Achromobacter xylosoxidans* (Amp/Sbt, Gen, Na, and Nit)* Serratia marcescens* (Cef and Nit),* Elizabethkingia meningoseptica* (Amp, Amp/Sbt, PpC/Tzba, Gen, and Nit), and* Pseudomonas fluorescens* (Amp, Amp/Sbt, Na, and Nit) (Table 2).

**Figure 1:**
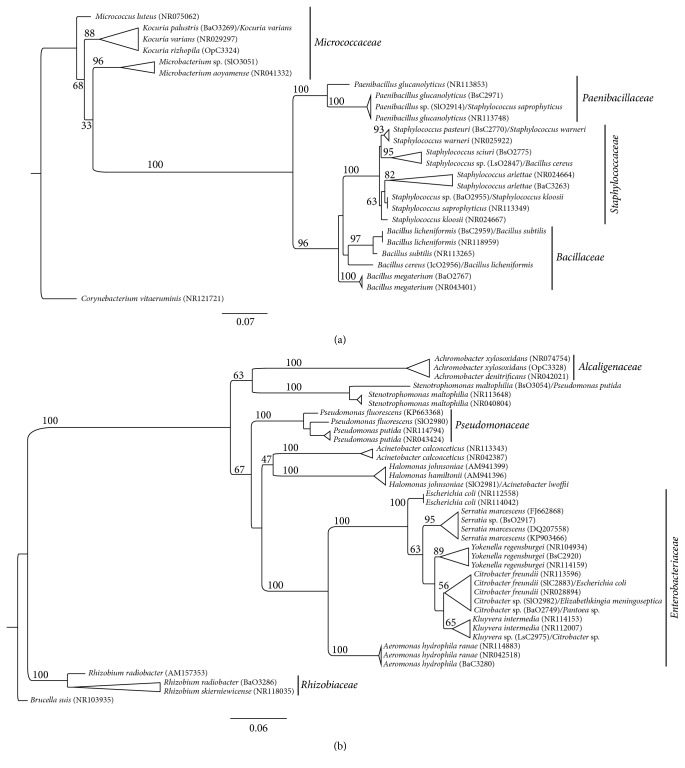
Phylogenetic position of local bacterial isolates by maximum-likelihood topology based on a partial sequence of the 16S ribosomal RNA gene. (a) Gram-positive tree, outgroup* Corynebacterium vitaeruminis*. (b) Gram-negative tree, outgroup* Brucella suis*. The first identity shown in each branch was obtained by the SeqMatch algorithm; the latter identity was obtained by VITEK biochemical analyses. Branches with only one identity stand for congruence between both SeqMatch and VITEK. In parentheses, there is our local isolate code or GenBank accession number.

In the phylogenetic structure obtained for Gram-positive bacteria, we observe clustering of four families: Micrococcaceae, Paenibacillaceae, Bacillaceae, and Staphylococcaceae. On this tree, conflict between identification analyses is shown for isolates SlO2914 and LsO2847. For the Gram-negative bacteria phylogenetic tree, our sequences clustered majorly in three families: Xanthomonadaceae, Pseudomonadaceae, and Enterobacteriaceae. In this topology, we have more taxonomic inconsistencies, mainly on the Enterobacteria, concerning these isolates: BsO3054, SlO2981, SlC2883, SlO2982, BaO2749, and LsC2975. However, more than 65 percent of the isolates analyzed with biochemical and molecular approach turned out in consistent identification at the genera level (Figure 1).

## 4. Discussion

Differences in habitat, predation strategies, and the type of prey can provide an explanation for the high variation in bacterial flora [30]. A marked trend on cloacal and oral isolates is not very well defined in our results. A factor that could influence the bacterial composition on oral or cloacal cavities is feeding habits. At the time of sampling, it is not known how recent has the snake eaten, which could explain why no differences on the number of isolates between terrestrial and arborous species were found. At the same time, it explains the lack of significant difference on oral and cloacal isolates. Another factor to consider is that snakes are very active and most species are not confined to a certain habitat [2].

Snake bites have a high rate of infection because of Gram-negative bacteria [31]. This is due to their eating habits, where the prey head is ingested first, leaving a colonization of fecal flora on the oral cavity. This also could explain the higher amount of enterobacterial isolates found in the mouth of the individuals sampled.

*Providencia *sp. was found in the oral cavity of captive snakes from Costa Rica [32]. Another study on* Bothrops jararaca *reported several species of bacteria from the oral cavity including* Providencia rettgeri, Staphylococcus aureus, Salmonella typhimurium, Citrobacter *sp., and* Morganella morganii *[33]. This finding coincided with our study, except that we found the last three genera on the cloaca not in the mouth. On a study carried out by Ferreira Junior et al. (2009) [34], they indicate the presence of* Salmonella enterica *and* M. morganii *in the oral cavity of rattlesnakes, and also* Citrobacter freundii *was found in the cloaca. In nonvenomous snakes, such as* Python regius *and* Clelia scyntalina, Serratia marcescens, M. morganii, and C. freundii *and other species in the oral cavity were identified [35]. On the other hand,* Elaphe quatuorlineata* (Colubridae) sampled at their natural habitats have shown bacterial isolates mainly of the genera* Serratia*,* Stenotrophomonas*,* Escherichia*,* Aeromonas*,* Pseudomonas*,* Staphylococcus,* and* Bacillus* [36].

Inconsistencies in bacterial identification between 16S rRNA sequencing and biochemical analyses conducted on the VITEK platform could be due to several factors. On a clinical study, 92% identity fidelity with a 16S sequencing approach was obtained, while VITEK only resolved 52% of the samples [37]. In our case, some isolates could be difficult to identify due to lack of entries in the database. However, in almost all of the samples, percentages of identification from VITEK are above 90%. Another factor that could be interfering is the lack of primer match suitable sites on the 16S rRNA for bacterial species level identification [38]; also the primers used (27f and 1492r) are widely known universal primers. This leads to a possible systematic underrepresentation of the matching phylogenetic group due to a difference in nucleotides [39].

Bacteria isolated from snakes on a zoo, such as* Citrobacter *sp.*, Enterobacter *sp.*, Escherichia coli, Hafnia alvei, Morganella morganii, Proteus *sp.*, Stenotrophomonas maltophilia*, and* Pseudomonas *sp., could be opportunistic pathogens and generate nosocomial infections. Besides,* Sphingomonas paucimobilis* has been associated with infections of the oral mucosa of humans [40]. On the other hand, pathogens like* Enterococcus faecalis, Salmonella arizonae,* and* Staphylococcus lentus* can generate zoonosis [12]. Similar bacterial genera were found compared to our results, where predominantly* Staphylococcus, Pseudomonas,* and* Enterobacter* match our findings [41].

Important to notice is the presence of* M. morganii, *a pathogen highly involved in abscess generation [30, 42]. The several species of* Staphylococcus *found can generate local infections and have been isolated in clinical cases [31].* Yokenella regensburgei *has been known to generate septicemias from soft tissue infections, especially for immunocompromised hosts [43]. The capacity of* Aeromonas hydrophila* is well known to cause severe infections after snakebites [44]. Another species identified was* Raoultella ornithinolytica*, a poorly described pathogen with rare cases of infection, with a high mortality rate reported (20%). This pathogen can produce bacteremia, skin infections, and respiratory infections [45].

Finally,* Elizabethkingia meningoseptica *was identified, an opportunistic pathogen that could have serious consequences on humans, with a reported 24% mortality rate over 118 patients [46]. Although it was found on a nonvenomous species, it is fairly commonly distributed [2]. This pathogen has been previously isolated in dead amphibians with cataracts and showed severe consequences to the host [47].

The vast majority of isolates showed antibiotic sensitivity patterns typical of wild, nonexposed strains and several natural resistance mechanisms widely distributed in nature. Resistance patterns suggested natural mechanisms of antibiotic resistance, such as constitutive chromosomal AmpC beta-lactamases and cephalosporinases, common in genera such as* Enterobacter*,* Citrobacter*,* Serratia*,* Proteus*,* Escherichia,* and* Morganella* [48]. Probable evidences of QNR protein and efflux pumps could be present in Enterobacteria and* Pseudomonas* strains, according to their resistance to nalidixic acid, but not to quinolones [49]. Similarly, resistance mechanisms to erythromycin and tetracycline due to efflux pumps could be present in Gram-positive cocci isolates, as they are widespread [50, 51].

An important diversity of aerobic bacteria was isolated (40 different bacterial species) from oral and cloacal mucous membrane from wildlife snakes species. Also with similar findings, other studies looked at bacterial diversity in different reptiles like turtles [52] and Komodo dragons [53], corroborating our results. Even studies regarding fungal diversity [44] conclude that these findings should be considering in the clinical picture when treating these animal bites. Importantly, antibiotics most appropriate in the case of infection by these pathogens are reported as well as the resistance found in these wild strains.

In summary, to our knowledge herein, this is the first report of a survey that combines biochemical and molecular approaches that identifies aerobic bacterial communities isolated from free-living venomous and nonvenomous snakes from Costa Rican rainforests. We also obtained an antibiotic susceptibility test (AST) for bacterial clusters inhabiting the cavities of local serpents. Our results revealed that the majority of the 12 bacterial families could bring health complications after a snakebite.

## Figures and Tables

**Table 1:** Bacteria isolates from oral and cloacal cavities of *Bothrops asper* (Ba), *Bothriechis schlegelii* (Bs), *Leptodeira septentrionalis* (Ls), *Sibon longifrenis* (Sl), *Oxyrhopus petolarius* (Op), *Oxybelis brevirostris* (Ob), and *Imantodes cenchoa* (Ic) analyzed and identified by VITEK biochemical approach and SeqMatch algorithm (RDP database). Isolates that were not sequenced are denoted with ND (no data).

Isolate	Bacterial identification (VITEK %)	SeqMatch identification (%)	Family
BaC3280	*Aeromonas hydrophila *(98)	*Aeromonas hydrophila *(100)	Aeromonadaceae
OpO3329	*Bordetella hinzii *(99)	ND	Alcaligenaceae
OpC3328	*Achromobacter xylosoxidans * (96)	*Achromobacter xylosoxidans * (94)	Alcaligenaceae
BaO2767	*Bacillus megaterium *(90)	*Bacillus megaterium *(100)	Bacillaceae
LsO2847	*Bacillus cereus *(97)	*Staphylococcus *sp. (87)	Bacillaceae
IcO2956	*Bacillus licheniformis *(89)	*Bacillus cereus* (100)	Bacillaceae
BsC2959	*Bacillus subtilis *(87)	*Bacillus licheniformis* (100)	Bacillaceae
IcC3322	*Bacillus mycoides *(95)	ND	Bacillaceae
BaO2749	*Pantoea *sp.(98)	*Citrobacter *sp. (96)	Enterobacteriaceae
SlC2883	*Escherichia coli *(88)	*Citrobacter freundii* (97)	Enterobacteriaceae
LsC2848	*Morganella morganii morganii* (99)	*Citrobacter *sp. (90)	Enterobacteriaceae
BsO2917	*Serratia marcescens *(99)	*Serratia *sp. (96)	Enterobacteriaceae
IcC2910	*Enterococcus faecalis *(99)	ND	Enterobacteriaceae
IcO2913	*Hafnia alvei *(99)	*ND*	Enterobacteriaceae
LsC2975	*Citrobacter freundii *(99)	*Kluyvera ascorbata* (98)	Enterobacteriaceae
BaO2743	*Providencia rettgeri *(99)	ND	Enterobacteriaceae
BaC2744	*Salmonella enterica diarizonae* (97)	*ND*	Enterobacteriaceae
BaC3287	*Serratia liquefaciens *(99)	ND	Enterobacteriaceae
BaC3290	*Citrobacter braakii *(99)	ND	Enterobacteriaceae
BsC2920	*Yokenella regensburgei *(95)	*Yokenella regensburgei *(86)	Enterobacteriaceae
IcC3357	*Proteus hauseri *(94)	ND	Enterobacteriaceae
OpC3327	*Raoultella ornithinolytica *(94)	ND	Enterobacteriaceae
SlO2982	*Elizabethkingia meningoseptica *(99)	*Citrobacter* sp. (95)	Flavobacteraceae
BsO3055	*Kocuria kristinae *(94)	ND	Micrococcaceae
SlO3051	*Micrococcus luteus *(97)	*Microbacterium *sp.(97)	Micrococcaceae
BaO3269	*Kocuria varians *(96)	*Kocuria palustris* (91)	Micrococcaceae
OpC3324	*Kocuria rhizophila *(99)	*Kocuria rhizophila *(95)	Micrococcaceae
SlO2981	*Acinetobacter lwoffii *(89)	*Halomonas johnsoniae* (94)	Moraxellaceae
BsC2971	*Paenibacillus glucanolyticus *(96)	*Paenibacillus glucanolyticus *(99)	Paenibacillaceae
SlO2980	*Pseudomonas fluorescens *(90)	*Pseudomonas fluorescens *(94)	Pseudomonaceae
BsO3054	*Pseudomonas putida *(99)	*Stenotrophomonas maltophilia *(75)	Pseudomonaceae
BaO3286	*Rhizobium radiobacter *(99)	*Rhizobium radiobacter *(91)	Rhizobiaceae
BaC3354	*Sphingomonas paucimobilis *(86)	ND	Sphingomonadaceae
SlO2914	*Staphylococcus saprophyticus *(99)	*Paenibacillus sp.* (98)	Staphylococcaceae
BaO2955	*Staphylococcus kloosii *(99)	*Staphylococcus sp.* (99)	Staphylococcaceae
SlC3049	*Staphylococcus xylosus *(91)	*Staphylococcus xylosus *(99)	Staphylococcaceae
BsO2775	*Staphylococcus sciuri *(97)	*Staphylococcus sciuri *(98)	Staphylococcaceae
BaC3263	*Staphylococcus arlettae *(99)	*Staphylococcus arlettae *(82)	Staphylococcaceae
BsC2770	*Staphylococcus warneri *(93)	*Staphylococcus pasteuri* (99)	Staphylococcaceae
SlC2885	*Stenotrophomonas maltophilia *(99)	ND	Xanthomonadaceae

**Table 2:** Antibiotic susceptibility testing (AST) of selected bacterial isolates from different free-living snakes species in Costa Rica.

Family	Bacterial specie	Amp	Amp/Sbt	Cip	Tet	PpC/Tzba	Cef	Rif	Cfa	Cftz	Cli	Cfe	Imi	Van	Mer	Min	Ami	Oxa	Gen	Qui/Da	Na	Eri	Mox	Lev	Cip2	Tei	Nit	Lin	Tri/Sul
Gram-positive																													
Bacillaceae	*Bacillus megaterium*	—	—	—	R	—	—	—	—	—	—	—	—	—	—	—	—	—	—	—	—	R	—	—	—	—	—	—	—
Staphylococcaceae	*Staphylococcus xylosus*	—	—	S	S	—	—	S	—	—	S	—	—	S	—	S	—	S	S	S	—	S	S	S	—	S	—	S	S
Staphylococcaceae	*Staphylococcus sciuri*	—	—	S	S	—	—	S	—	—	S	—	—	S	—	S	—	S	S	S	—	S	S	S	—	S	S	S	S
Staphylococcaceae	*Staphylococcus kloosii*	—	—	—	S	—	—	—	—	—	S	—	—	—	—	—	—	S	S	S	—	S	—	—	—	—	S	—	S
Staphylococcaceae	*Staphylococcus warneri*	—	—	—	S	—	—	—	—	—	R	—	—	—	—	—	—	S	S	R	—	R	—	—	—	—	S	—	S

Gram-negative																													
Aeromonadaceae	*Aeromonas hydrophila*	R	R	—	—	S	—	—	S	S	—	—	S	—	S	—	S	—	S	—	S	—	—	—	S	—	S	—	S
Alcaligenaceae	*Achromobacter xylosoxidans*	S	I	—	—	S	—	—	—	S	—	—	S	—	S	—	S	—	I	—	R	—	—	—	S	—	R	—	S
Enterobacteriaceae	*Providencia rettgeri*	R	S	—	—	S	R	—	S	S	—	S	S	—	S	—	S	—	S	—	S	—	—	—	S	—	R	—	S
Enterobacteriaceae	*Citrobacter freundii*	—	—	—	—	S	R	—	S	S	—	S	S	—	S	—	S	—	S	—	R	—	—	—	S	—	S	—	S
Enterobacteriaceae	*Proteus hauseri*	R	S	—	—	S	R	—	S	S	—	S	S	—	S	—	S	—	S	—	S	—	—	—	S	—	R	—	S
Enterobacteriaceae	*Serratia marcescens*	—	—	—	—	—	R	—	S	S	—	S		—	S	—	S	—	S	—	S	—	—	—	S	—	R	—	S
Enterobacteriaceae	*Serratia liquefaciens*	—	—	—	—	S	R	—	S	S	—	S	S	—	S	—	S	—	S	—	S	—	—	—	S	—	R	—	S
Enterobacteriaceae	*Morganella morganii *ssp.* morganii*	R	S	—	—	S	R	—	I	S	—	S	S	—	S	—	S	—	S	—	S	—	—	—	S	—	R	—	R
Enterobacteriaceae	*Citrobacter braakii*	—	—	—	—	S	R	—	S	S	—	S	S	—	S	—	S	—	S	—	S	—	—	—	S	—	S	—	S
Enterobacteriaceae	*Raoultella ornithinolytica*	R	S	—	—	S	S	—	S	S	—	S	S	—	S	—	S	—	S	—	S	—	—	—	S	—	S	—	S
Enterobacteriaceae	*Escherichia coli*	S	S	—	—	S	—	—	—	—	—	—	—	—	—	—	—	—	S	—	R	—	—	—	—	—	S	—	S
Enterobacteriaceae	*Enterococcus faecalis*	R	—	—	S	—	—	—	—	—	—	—	—	—	—	—	—	—	—	R	—	—	—	—	—	—	—	—	R
Enterobacteriaceae	*Hafnia alvei*	R	—	—	R	R	R	—	—	—	—	—	—	—	—	—	—	—	—	—	S	—	—	—	—	—	S	—	S
Flavobacteriaceae	*Elizabethkingia meningoseptica*	R	R	—	—	R	R	—	—	—	—	—	R	—	R	—	R	—	R	—	S	—	—	—	—	—	R	—	S
Moraxellaceae	*Acinetobacter lwoffii*	S	S	—	—	—	—	—	—	—	—	—		—	—	—	—	—	—	—	S	—	—	—	—	—	—	—	—
Pseudomonaceae	*Pseudomonas putida*	R	R	—	—	—	—	—	I	S	—	—	S	—	S	—	S	—	S	—	R	—	—	—	S	—	R	—	R
Pseudomonaceae	*Pseudomonas fluorescens*	R	R	—	—	—	—	—	—	—	—	—	—	—	—	—	—	—	—	—	R	—	—	—	—	—	R	—	—
Xanthomonadaceae	*Stenotrophomonas maltophilia*	—	—	—	—	—	—	—	—	—	—	—	—	—	—	—	—	—	—	—	—	—	—	—	—	—	—	—	S

R: resistant, S: sensitive, I: moderately sensitive, and —: no data. Am: ampicillin; Amp/Sbt: ampicillin/sulbactam; Cip: ciprofloxacin; Tet: tetracycline; PpC/Tzba: piperacillin/tazobactam; Cef: cephalothin; Rif: rifampin; Cfa, cefotaxime; Cft, ceftazidime; Cli, clindamycin; Cfe, cefepime; Imi: imipenem; Van: vancomycin; Mer: meropenem; Min: minocycline; Ami: amikacin; Oxa: oxacillin; Gen: gentamicin; Qui/Da: quinupristin/dalfopristin; Na: nalidixic acid; Er: erythromycin; Mox: moxifloxacin; Lev: levofloxacin; Cip2: ciprofloxacin 2; Tei: teicoplanin; Nit: nitrofurantoin; Lin: linezolid; Tri/Sul: trimethoprim/sulfamethoxazole.
